# Efficacy and safety of different molecular targeted agents based on chemotherapy for gastric cancer patients treatment: a network meta-analysis

**DOI:** 10.18632/oncotarget.17192

**Published:** 2017-04-18

**Authors:** Zheng Ren, Jinping Sun, Xinfang Sun, Hongtao Hou, Ke Li, Quanxing Ge

**Affiliations:** ^1^ Department of Digestive Internal Medicine, Huaihe Hospital of Henan University, Kaifeng 475000, Henan, China

**Keywords:** gastric cancer, molecular targeted agents, chemotherapy, network meta-analysis, efficacy

## Abstract

Increasing numbers of reports have been published to demonstrate that molecular targeted agents are able to improve the efficacy of chemotherapy in gastric cancer. This network meta-analysis aimed to evaluate the efficacy and safety of different molecular targeted agents, which were divided into six groups based on the targets including hepatocyte growth factor receptor (c-MET), vascular endothelial factor and its receptor (VEGF/VEGFR), human epidermal growth factor receptor 2 (HER2), epidermal growth factor receptor (EGFR), mammalian target of rapamycin (mTOR) and tyrosine kinase inhibitor (TKI). These six groups of targeted agents were evaluated for their efficacy outcomes measured by overall survival (OS), progression-free survival (PFS) and overall response rate (ORR). While their safety was measured 7 adverse events, including fatigue, anaemia, vomiting, neutropenia, thrombocytopenia, diarrhea and nausea. A total of 23 articles were included after extensive searching and strict inclusion, HER2 and VEGF(R) turned out to be the two most effective targeted drugs for their outstanding performance in OS and PFS. However, they were associated with severe adverse events, including fatigue, neutropenia and diarrhea. Therefore, they should be used with caution during their application. In conclusion, VEGF(R) and HER2 have the potential to be the optimal target agents for their survival efficacy, while the adverse events associated with them should be paid attention in application.

## INTRODUCTION

Gastric cancer (GC), also known as stomach cancer, is a malignant tumor caused by environmental and genetic factors. Although the incidence and mortality of GC have extremely decreased since the 21st century, it is still the fourth major cause of cancer deaths [1]. In 2016, the number of expected new cases is 26,370 in US, with 10,730 expected deaths [2]. The epidemic nature and high mortality of GC have driven researchers to assess the relative efficacy of various treatments. Surgery has been identified to be curative for early stage patients, and preoperative or postoperative chemotherapy and chemoradiotherapy can improve the remedy [3]. However, about 60% of the patients are diagnosed after 65 years old, when surgery is considered to be risky [4]. Thus, chemotherapy is widely used as a major treatment for its applicability to almost all patients.

Recently, it was reported that molecular targeted agents are able to significantly reinforce the efficacy of chemotherapy [5]. Several molecular targets and medications have been researched and combined with chemotherapy. One group of target molecules includes vascular endothelial growth factor (VEGF) and vascular endothelial factor receptor (VEGFR), both of which facilitates in the angiogenesis and metastasis of GC [6]. Another group of targets is related to epidermis. For instance, human epidermal growth factor receptor 2 (HER2) could stimulate the proliferation of tumor cells, and its overexpression triggers the development of cancer [7]. Epidermal growth factor receptor (EGFR) could also activate the macrophage in nonspecific immunity [8]. In addition, the mammalian target of rapamycin (mTOR) contributed to cellular apoptosis and proliferation [2]. Tyrosine kinase inhibitor (TKI) prohibits the expression of tyrosine kinase in order to stop the signal transduction for proteins [9] Hepatocyte growth factor receptor (HGFR) is encoded by c-MET gene and accelerates epithelial–mesenchymal transition [2]. By using these target agents in chemotherapy, the efficacy could be multiplied.

Several reports have been published to illustrate the efficacy of molecular targeted treatment. A study in Japan reported that the use of Bevacizumab, an antibody of VEGF, could significantly increase progression-free survival (PFS). Ramucirumab, an antibody of VEGFR, was proved to be able to increase the overall survival (OS) rate in combination with paclitaxel [10]. TKI plus docetaxel would promote the overall response rate (ORR) compared to the monotherapy of docetaxel [11]. Although EGFR and mTOR targeted agents did not seem to significantly improve the effect of chemotherapy, these agents provided a new method for GC treatment. However, despite desirable results targeted therapies may bring, they were also associated with adverse events. According to the US National Cancer Institute (NCI), these alternative treatments may produce grade 3–4 adverse events [12]. Hence, in order to amplify the efficacy of chemotherapy while reduce severe adverse events as many as possible, this network meta-analysis (NMA) was designed to compare the relative efficacy and safety of different targeted agents in combination with chemotherapy and aimed to select an optimal targeted drug.

## RESULTS

### Study characteristics

**Figure 1:**
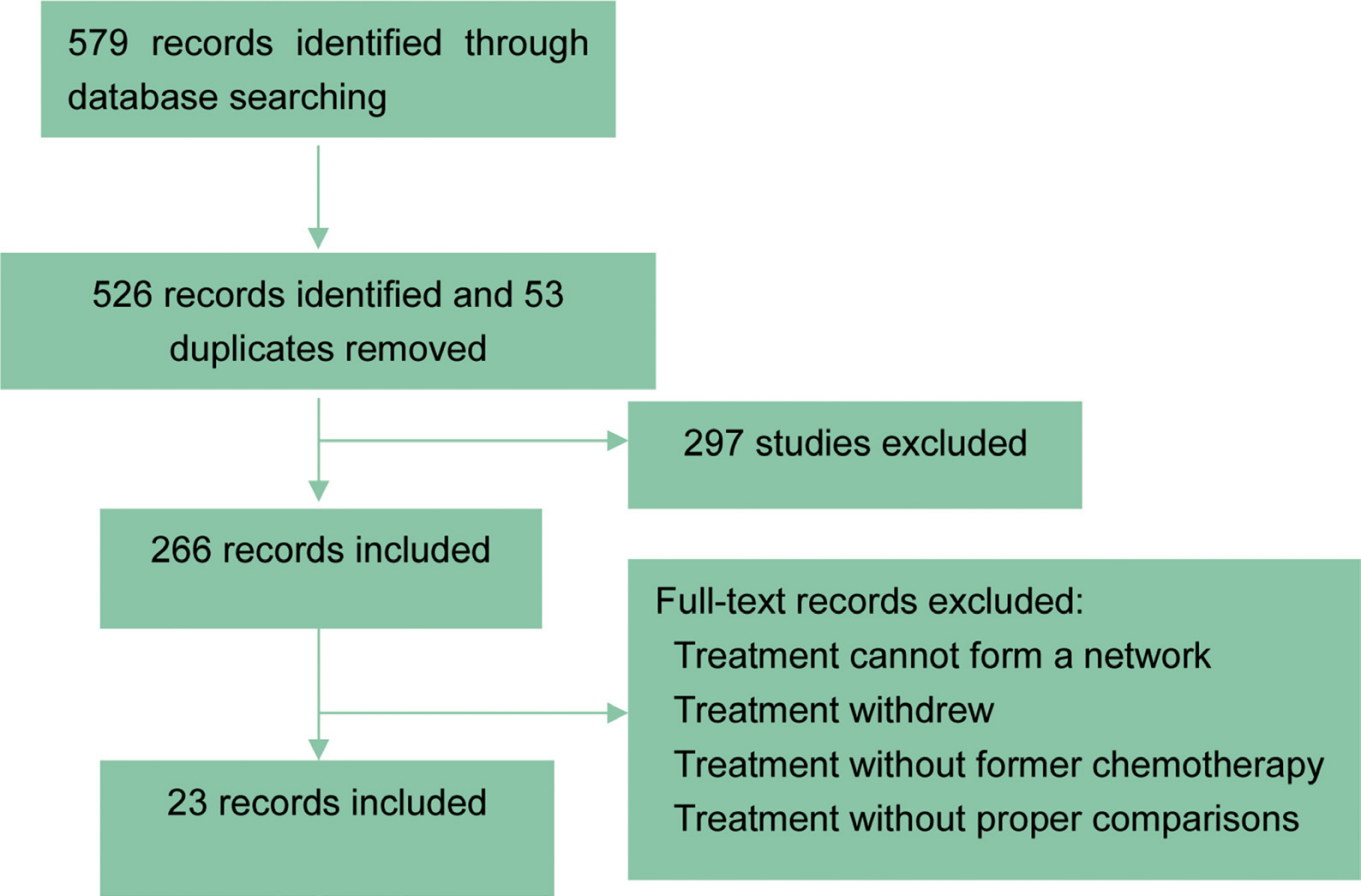
Flow charts of included studies The searching and exclusion process was illustrated.

**Figure 2:**
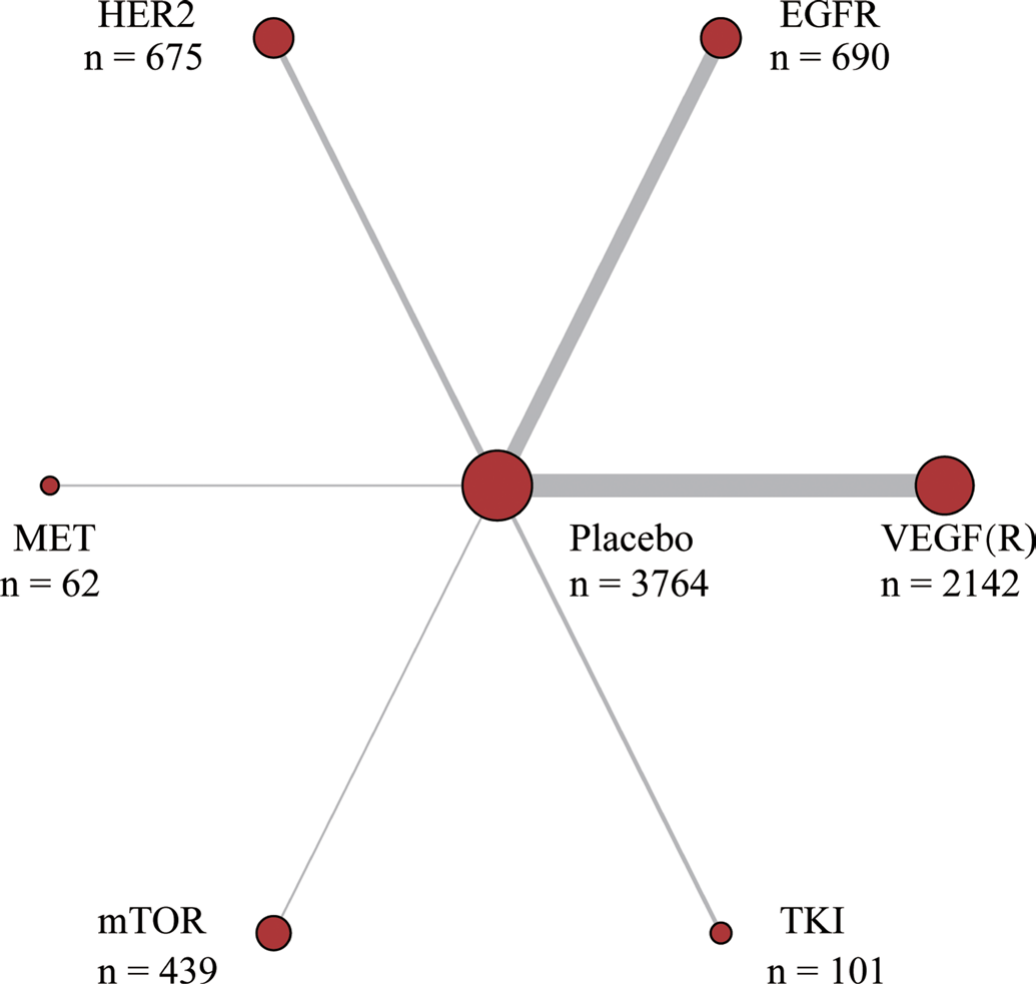
Network plot of molecular targeted agents for gastric cancer The width of the lines is proportional to the number of trials comparing each pair of treatments target; the area of circles represents the cumulative number of patients for each intervention.

As shown in Figure 1, through extensive searching and exclusion, 23 articles with 4,109 patients were finally obtained [1–3, 11, 13–31]. The baseline characteristics of all included studies shown presented in Table 1. All the 23 studies involved patients with advanced cancer, while some with metastatic cancer or unresectable caner. 12 of the studies were associated with first-line chemotherapy while 8 of them were associated with second-line chemotherapy. All the studies compared targeted agents with placebo (in combination with chemotherapy), with 11 for anti-VEGFR, 7 for anti-EGFR, 3 for anti-HER2, 2 included TKI, 1 for anti-mTOR and another one for anti-MET (Figure 2).

**Table 1:** Main characteristics of included studies

Study information	Follow-up (months)	Stage	Chemotherapy	Experiment group				Placebo group		Outcomes
				Size	Age (range)	Target	Regimen	Size	Age (range)	
Bang, 2010, Korea	34	Advanced	First-line	294	59	HER2	Trastuzumab	290	59	①②③④⑤⑥⑦⑦⑨⑩
Casak, 2015, USA	30	Metastatic/unresectable	First-line	327	-	VEGF(R)	Ramucirumab	329	-	①⑤⑥⑦⑦⑨
Du, 2015, China	60	Advanced/metastatic	First-line	31	48(22-75)	EGFR	Nimotuzumab	31	53(30-76)	①②③④⑤⑥⑦⑦⑨⑩
Fuchs, 2014, USA	28	Advanced	First-line	238	60(52-67)	VEGF(R)	Ramucirumab	117	60(51-71)	①②③⑤⑥⑦
Hecht, 2016, USA	45	Advanced/metastatic	-	249	61(19-86)	HER2	Lapatinib	238	59(27-84)	①②③④⑤⑥⑦⑦⑨
Lordick, 2013, Germany	42	Advanced/metastatic	First-line	455	60(23-84)	EGFR	Cetuximab	449	59(18-81)	①②③④⑤⑥⑦⑦⑨⑩
Manish, 2016, Multicenter	12	Advanced	First-line	62	59(33-78)	MET	Onartuzumab	61	57(31-82)	①②③⑨⑩
Markus, 2016, Germany	12	Advanced	Second-line	45	62(37-76)	TKI	Sunitinib	45	57(28-84)	①②③④⑤⑥⑦⑨
Muro, 2016, Japan	28	Advanced	Second-line	109	62(28-76)	VEGF(R)	Ramucirumab	114	62(28-81)	①②③④⑤⑥⑦⑦⑨⑩
				221	60(25-83)	VEGF(R)	Ramucirumab	221	60(24-84)	①②③④⑤⑥⑦⑦⑨⑩
Ohtsu, 2011, Japan	24	Advanced	First-line	387	58(22-81)	VEGF(R)	Bevacizumab	387	59(22-82)	①②③④⑥⑦⑦⑨
Ohtsu, 2013, Japan	24	Advanced	Second-line	439	62(20-86)	mTOR	Everolimus	217	62(26-88)	①②③④⑤⑥⑦⑦⑨⑩
Rao, 2010, UK	24	Advanced	First-line	35	59(29-79)	EGFR	Matuzumab	36	64(36-76)	①②③④⑤⑥⑦⑦⑨⑩
Richards, 2013, USA	36	Advanced	First-line	75	64(25-84)	EGFR	Cetuximab	75	62(25-82)	①②③⑤⑦⑨⑩
Satoh, 2015, Japan	20	Advanced	Second-line	40	60	EGFR	Nimotuzumab	42	64	①②④⑤⑥⑦⑦⑨⑩
Satoh, 2014, Japan	45	Advanced	Second-line	132	61(32-79)	HER2	Lapatinib	129	62(22-80)	①②③④⑤⑥⑦⑦⑨
Shen, 2015, China	27	Advanced/metastatic	First-line	100	-	VEGF(R)	Bevacizumab	102	-	①②③④⑥⑦⑦⑨⑩
Shitara, 2016, Japan	28	Advanced/metastatic	Second-line	68	64(34-76)	VEGF(R)	Ramucirumab	72	64.5(29-76)	①②③⑤⑥⑦⑨
		/unresectable		198	60(25-83)	VEGF(R)	Ramucirumab	200	61(24-84)	①②③⑤⑥⑦⑨
Tebbutt, 2016, Australia	18	Advanced	First-line	37	64(40-79)	EGFR	Panitumumab	39	59(37-77)	①②④⑤⑥⑦⑨
Wilke, 2014, Germany	28	Advanced	Second-line	330	61(25-83)	VEGF(R)	Ramucirumab	335	61(24-84)	①②③④⑤⑥⑦⑦⑨⑩
Xu, 2013, China	30	Advanced	-	80	-	VEGF(R)	Endostar	85	-	②③④⑦⑦⑨⑩
Xu, 2014, China	15	Advanced	-	17	-	EGFR	Nimotuzumab	17	-	①②③
Yi, 2012, Korea	24	Advanced	Second-line	56	54(20-72)	TKI	Sunitinib	49	52(36-70)	①②③⑦⑦⑨⑩
Yoon, 2016, USA	17	Advanced	First-line	84	65(27-83)	VEGF(R)	Ramucirumab	84	64(34-82)	①②③④⑤⑦⑨⑩

Note: ①, overall survival; ② progression-free survival; ③, overall response rate; ④, Fatigue; ⑤, Anaemia; ⑥, Neutropenia; ⑦, Vomiting; ⑧, Diarrhoea; ⑨, Nausea; ⑩, Thrombocypenia; Abbreviation: HER2, Human epidermal growth factor receptor-2; EGFR, epidermal growth factor receptor; VEGFR, vascular endothelial growth factor receptor; VEGF, vascular endothelial growth factor; TKI, tyrosine kinase inhibitor; mTOR, mammalian target of rapamycin; HGRF, Hepatocyte growth factor receptor

### Survival outcomes

**Figure 3:**
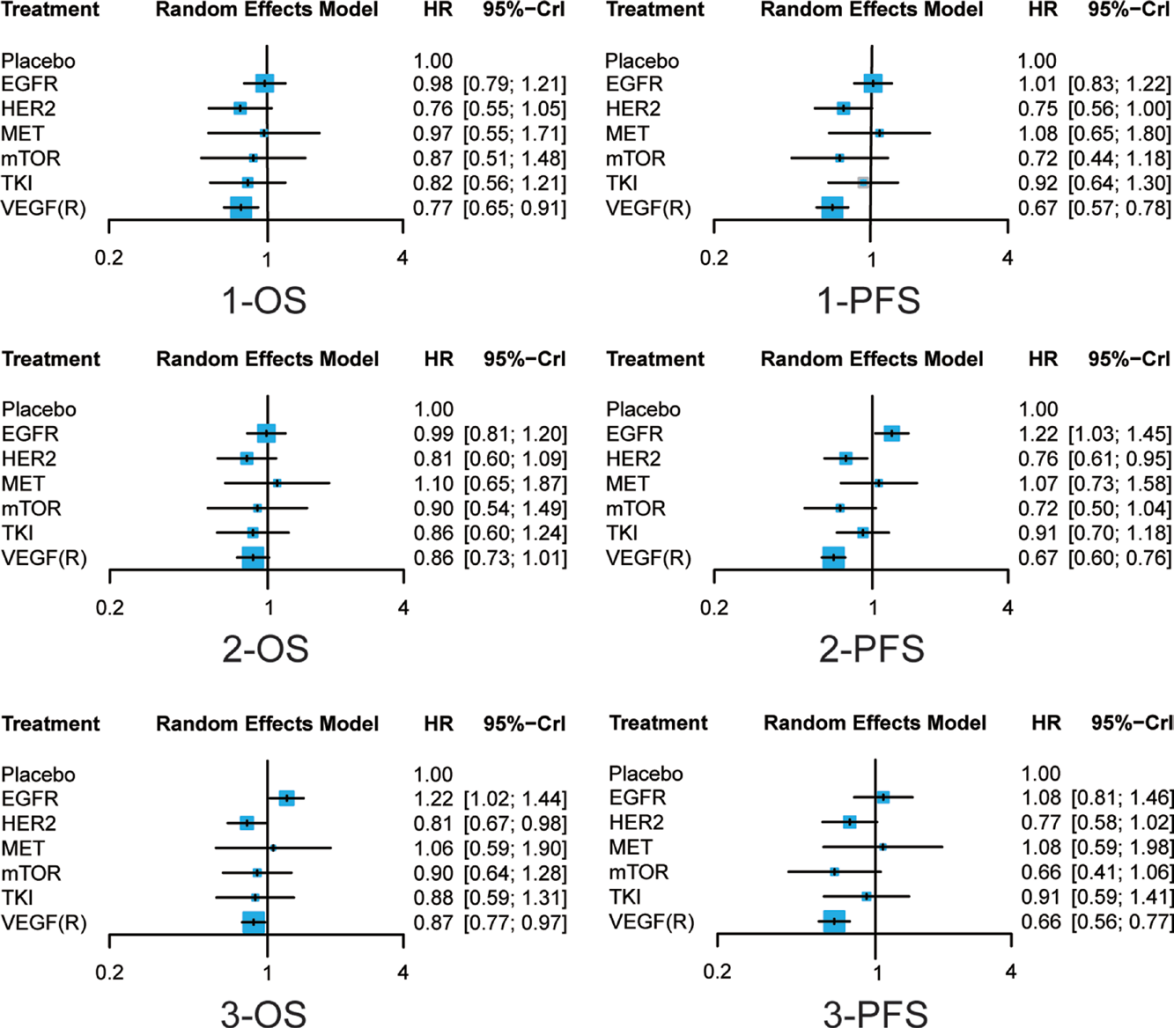
Forest plots of survival outcomes of different treatments Hazard ratios (HRs) with corresponding 95% credible intervals (95% CrI) were used to measure the relative efficacy of different treatments

Results of survival outcomes were shown in Table 2 and Figure 3. For one-year OS (1-OS), only VEFG(R) was superior to placebo (HR = 0.77, 95% CrI = 0.65–0.91). While for one-year PFS (1-PFS), both HER2 and VEGF(R) were more effective than placebo (HR = 0.75, 95% CrI = 0.56–1.00; HR = 0.67, 95% CrI = 0.57–0.78, respectively). In the meantime, VEGF(R) exhibited better performance than EGFR (HR = 0.66, 95% CrI = 0.51–0.85). No significantly statistical difference was found in 2-OS, but in terms of 2-PFS, there was a big difference of efficacy among different drugs. Similar to 1-PFS, both HER2 and VEGF(R) were more effective than placebo (HR = 0.76, 95% CrI = 0.61–0.95; HR = 0.67, 95% CrI = 0.60–0.76, respectively), however, EGFR was the only one inferior to placebo (HR = 1.22, 95% CrI = 1.03–1.45), and exhibited less satisfying results compared to HER2 (HR = 0.63, 95% CrI = 0.47–0.82), m-TOR(HR = 0.59, 95% CrI = 0.40–0.88) and VEGF(R) (HR = 0.55, 95% CrI = 0.45–0.68). Plus, VEGF(R) was more effective than MET (HR = 0.63, 95% CrI = 0.42–0.95) and TKI (HR = 0.74, 95% CrI = 0.56–0.99). Similar results repeated with respect to 3-OS, with EGFR inferior to placebo (HR = 1.22, 95% CrI = 1.02–1.44), HER2 (HR = 1.50, 95% CrI = 1.16–1.95) and VEGF(R) (HR = 1.40, 95% CrI = 1.14–1.73), while HER2 and VEGF(R) superior to placebo (HR = 0.81, 95% CrI = 0.67–0.98; HR = 0.87, 95% CrI = 0.77–0.97).

**Table 2:** Network meta-analysis results of survival outcomes

**1-OS**	**Placebo**	1.01 (0.83,1.22)	0.75 (0.56,1.00)	1.08 (0.65,1.80)	0.72 (0.44,1.18)	0.92 (0.64,1.30)	0.67 (0.57,0.78)	**1-PFS**
	0.98 (0.79,1.21)	**EGFR**	0.74 (0.52,1.05)	1.07 (0.62,1.85)	0.71 (0.42,1.21)	0.91 (0.61,1.35)	0.66 (0.51,0.85)	
	0.76 (0.55,1.05)	0.78 (0.53,1.14)	**HER2**	1.44 (0.80,2.60)	0.96 (0.54,1.70)	1.22 (0.77,1.93)	0.89 (0.64,1.25)	
	0.97 (0.55,1.71)	0.99 (0.54,1.81)	1.27 (0.66,2.44)	MET	0.67 (0.33,1.36)	0.85 (0.45,1.58)	0.62 (0.36,1.06)	
	0.87 (0.51,1.48)	0.89 (0.50,1.57)	1.14 (0.62,2.12)	0.90 (0.41,1.95)	**mTOR**	1.27 (0.69,2.33)	0.93 (0.55,1.56)	
	0.82 (0.56,1.21)	0.84 (0.54,1.30)	1.08 (0.66,1.78)	0.85 (0.43,1.68)	0.95 (0.49,1.82)	**TKI**	0.73 (0.49,1.08)	
	0.77 (0.65,0.91)	0.79 (0.60,1.03)	1.01 (0.70,1.45)	0.79 (0.44,1.43)	0.88 (0.51,1.54)	0.93 (0.61,1.42)	**VEGF (R)**	
**2-OS**	**Placebo**	1.22 (1.03,1.45)	0.76 (0.61,0.95)	1.07 (0.73,1.58)	0.72 (0.50,1.04)	0.91 (0.70,1.18)	0.67 (0.60,0.76)	**2-PFS**
	0.99 (0.81,1.20)	EGFR	0.63 (0.47,0.82)	0.88 (0.57,1.34)	0.59 (0.40,0.88)	0.74 (0.54,1.02)	0.55 (0.45,0.68)	
	0.81 (0.60,1.09)	0.82 (0.57,1.17)	**HER2**	1.40 (0.90,2.18)	0.94 (0.62,1.44)	1.19 (0.85,1.68)	0.88 (0.69,1.13)	
	1.10 (0.65,1.87)	1.12 (0.64,1.96)	1.36 (0.74,2.50)	**MET**	0.67 (0.40,1.14)	0.85 (0.53,1.36)	0.63 (0.42,0.95)	
	0.90 (0.54,1.49)	0.91 (0.53,1.57)	1.11 (0.62,2.00)	0.82 (0.39,1.70)	**mTOR**	1.26 (0.81,1.97)	0.94 (0.64,1.37)	
	0.86 (0.60,1.24)	0.87 (0.58,1.32)	1.06 (0.66,1.70)	0.78 (0.41,1.48)	0.95 (0.51,1.78)	**TKI**	0.74 (0.56,0.99)	
	0.86 (0.73,1.01)	0.87 (0.68,1.13)	1.06 (0.76,1.49)	0.78 (0.45,1.36)	0.96 (0.56,1.63)	1.00 (0.67,1.49)	**VEGF (R)**	
**3-OS**	**Placebo**	1.08 (0.81,1.46)	0.77 (0.58,1.02)	1.08 (0.59,1.98)	0.66 (0.41,1.06)	0.91 (0.59,1.41)	0.66 (0.56,0.77)	**3-PFS**
	1.22 (1.02,1.44)	**EGFR**	0.71 (0.47,1.07)	1.00 (0.51,1.95)	0.61 (0.35,1.06)	0.84 (0.50,1.43)	0.61 (0.43,0.85)	
	0.81 (0.67,0.98)	0.67 (0.51,0.86)	**HER2**	1.40 (0.72,2.73)	0.86 (0.50,1.48)	1.19 (0.71,1.98)	0.85 (0.62,1.17)	
	1.06 (0.59,1.90)	0.87 (0.47,1.60)	1.31 (0.71,2.42)	**MET**	0.61 (0.28,1.31)	0.85 (0.40,1.78)	0.61 (0.33,1.14)	
	0.90 (0.64,1.28)	0.74 (0.50,1.09)	1.11 (0.74,1.66)	0.85 (0.43,1.68)	**mTOR**	1.39 (0.73,2.62)	1.00 (0.61,1.64)	
	0.88 (0.59,1.31)	0.72 (0.47,1.11)	1.09 (0.70,1.69)	0.83 (0.41,1.68)	0.98 (0.58,1.66)	**TKI**	0.72 (0.45,1.14)	
	0.87 (0.77,0.97)	0.71 (0.58,0.88)	1.07 (0.85,1.34)	0.82 (0.45,1.48)	0.96 (0.67,1.39)	0.98 (0.65,1.49)	**VEGF (R)**	

Abbreviations: HER2, Human epidermal growth factor receptor-2; EGFR, epidermal growth factor receptor; VEGF(R), vascular endothelial growth factor (receptor); TKI, tyrosine kinase inhibitor; mTOR, mammalian target of rapamycin; HGRF, Hepatocyte growth factor receptor.

### Response rate and adverse events (grade ≥ 3)

**Figure 4:**
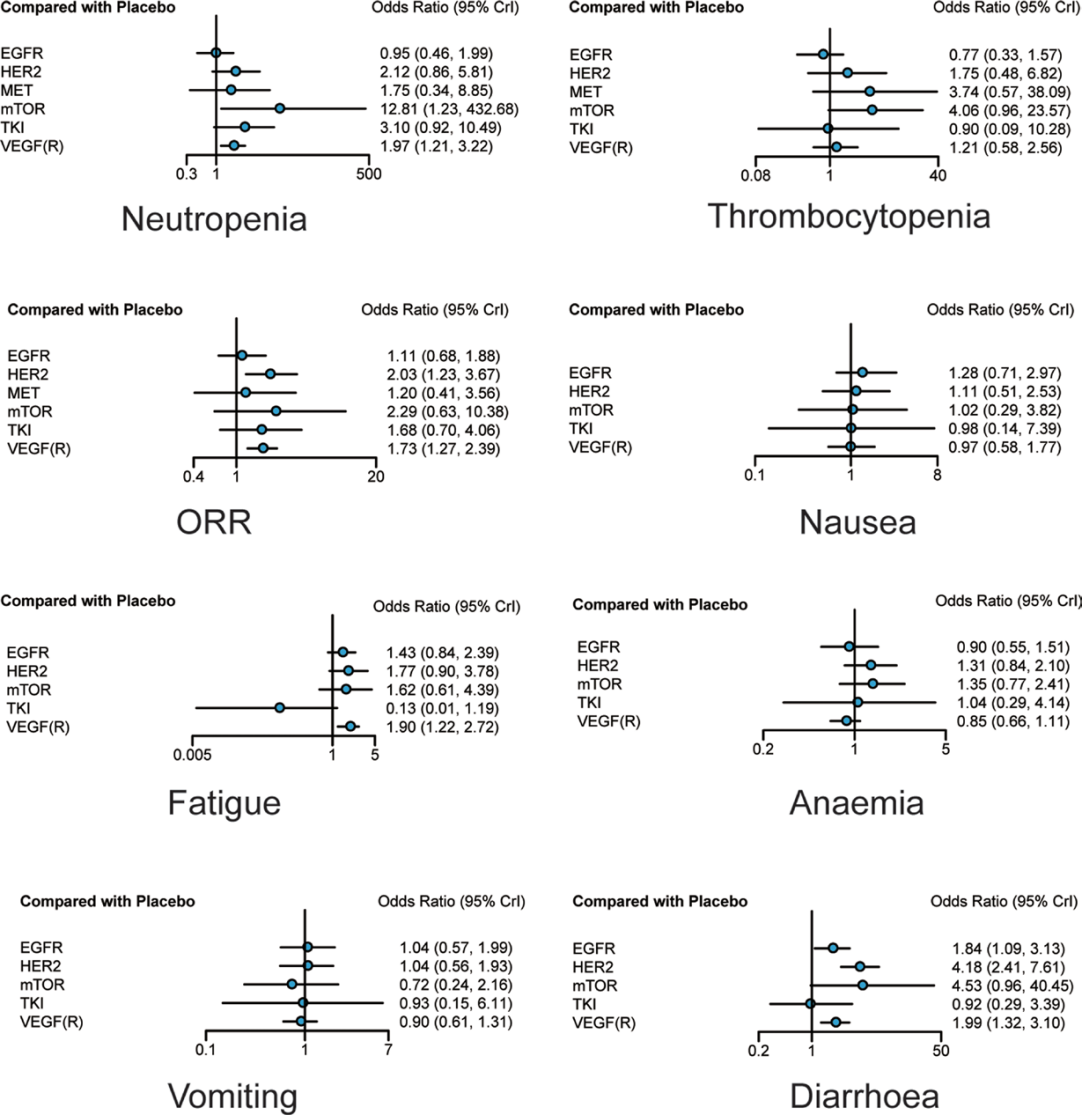
Forest plots of overall response rate and adverse events of different treatments Odds ratios (ORs) with corresponding 95% credible intervals were used to measure the relative efficacy and safety of different treatments.

Results of response rate and adverse events were exhibited in Table 3 and Figure 4. Consistent with the results with respect to survival, HER2 and VEGF(R) were the only two agents that brought higher response rate among patients compared with placebo (OR = 2.03, 95% CrI = 1.23–3.67; OR = 1.73, 95% CrI = 1.27–2.39, respectively). As for adverse events, no significant statistical difference was detected in terms of vomiting, anaemia and nausea. M-TOR invited more thrombocytopenia events than EGFR (OR = 5.37, 95% CrI = 1.11–37.71) and was associated with higher risk of neutropenia compared to placebo and EGFR (OR = 12.81, 95% CrI = 1.23–432.68; OR = 13.46, 95% CrI = 1.13–487.85, respectively). VEGF(R) introduced more neutropenia events and fatigue events than placebo (OR = 1.97, 95% CrI = 1.21–3.22; OR = 1.90, 95% CrI = 1.22–2.72), while TKI seemed to reduce the risk of fatigue for its superiority to all other drugs. In terms of diarrhea, EGFR, HER2 and VEGF(R) were associated with higher risk compared to placebo (OR = 1.84, 95% CrI = 1.09–3.13; OR = 4.18, 95% CrI = 2.41–7.61; OR = 1.99, 95% CrI = 1.32–3.10, respectively), and moreover, HER2 seemed to bring more diarrhea events than EGFR, TKI and VEGF(R) (OR = 2.25, 95% CrI = 1.07–5.21; OR = 4.48, 95% CrI = 1.11–17.46; OR = 2.08, 95% CrI = 1.04–4.26).

**Table 3:** Network meta-analysis results of response rate and adverse events

**ORR**	**Placebo**	1.28 (0.71,2.97)	1.11 (0.51,2.53)	1.02 (0.29,3.82)	0.98 (0.14,7.39)	0.97 (0.58,1.77)	-	**Nausea**
	1.11 (0.68,1.88)	EGFR	0.86 (0.27,2.25)	0.79 (0.16,3.16)	0.76 (0.09,5.99)	0.75 (0.29,1.67)	-	
	2.03 (1.23,3.67)	1.84 (0.90,3.90)	HER2	0.92 (0.20,4.26)	0.89 (0.11,7.46)	0.87 (0.34,2.36)	-	
	2.29 (0.63,10.38)	2.08 (0.51,9.97)	1.12 (0.27,5.47)	mTOR	0.96 (0.09,10.80)	0.94 (0.24,3.97)	-	
	1.68 (0.70,4.06)	1.54 (0.54,4.10)	0.83 (0.29,2.25)	0.73 (0.13,3.53)	TKI	1.00 (0.13,7.77)	-	
	1.73 (1.27,2.39)	1.57 (0.85,2.80)	0.85 (0.44,1.55)	0.76 (0.16,2.89)	1.03 (0.41,2.59)	VEGF (R)	-	
	1.20 (0.41,3.56)	1.08 (0.33,3.53)	0.58 (0.17,1.93)	0.52 (0.08,2.86)	0.71 (0.18,2.83)	0.69 (0.23,2.14)	MET	
Neutropenia	Placebo	0.77 (0.33,1.57)	1.75 (0.48,6.82)	3.74 (0.57,38.09)	4.06 (0.96,23.57)	0.90 (0.09,10.28)	1.21 (0.58,2.56)	Thrombocytopenia
	0.95 (0.46,1.99)	EGFR	2.29 (0.54,11.94)	5.00 (0.63,59.15)	5.37 (1.11,37.71)	1.20 (0.11,15.80)	1.58 (0.57,4.95)	
	2.12 (0.86,5.81)	2.23 (0.70,7.69)	HER2	2.14 (0.21,31.19)	2.34 (0.32,20.91)	0.51 (0.03,7.85)	0.68 (0.14,3.10)	
	1.75 (0.34,8.85)	1.84 (0.31,10.70)	0.83 (0.12,5.16)	MET	1.08 (0.07,14.01)	0.23 (0.01,5.58)	0.32 (0.03,2.44)	
	12.81 (1.23,432.68)	13.46 (1.13,487.85)	6.05 (0.45,223.63)	7.54 (0.41,347.23)	mTOR	0.21 (0.01,3.67)	0.30 (0.04,1.49)	
	3.10 (0.92,10.49)	3.25 (0.79,13.20)	1.46 (0.30,6.62)	1.77 (0.23,13.33)	0.24 (0.01,3.53)	TKI	1.34 (0.10,15.18)	
	1.97 (1.21,3.22)	2.08 (0.85,4.95)	0.94 (0.30,2.59)	1.13 (0.21,6.17)	0.15 (0.00,1.73)	0.64 (0.17,2.36)	VEGF (R)	
Fatigue	Placebo	0.90 (0.55,1.51)	1.31 (0.84,2.10)	1.35 (0.77,2.41)	1.04 (0.29,4.14)	0.85 (0.66,1.11)		Anaemia
	1.43 (0.84,2.39)	EGFR	1.46 (0.73,2.86)	1.51 (0.70,3.16)	1.16 (0.29,5.26)	0.95 (0.53,1.68)	
	1.77 (0.90,3.78)	1.22 (0.53,3.16)	HER2	1.04 (0.50,2.16)	0.79 (0.20,3.46)	0.65 (0.38,1.09)	
	1.62 (0.61,4.39)	1.13 (0.38,3.49)	0.91 (0.26,3.03)	mTOR	0.77 (0.19,3.22)	0.63 (0.34,1.16)	
	0.13 (0.01,1.19)	0.09 (0.00,0.86)	0.07 (0.00,0.77)	0.08 (0.00,0.96)	TKI	0.83 (0.20,3.06)	
	1.90 (1.22,2.72)	1.32 (0.67,2.48)	1.07 (0.43,2.29)	1.19 (0.38,3.16)	14.44 (1.51,333.62)	VEGF (R)	
Vomiting	Placebo	1.84 (1.09,3.13)	4.18 (2.41,7.61)	4.53 (0.96,40.45)	0.92 (0.29,3.39)	1.99 (1.32,3.10)		Diarrhoea
	1.04 (0.57,1.99)	EGFR	2.25 (1.07,5.21)	2.44 (0.47,22.87)	0.51 (0.14,1.97)	1.08 (0.57,2.16)	
	1.04 (0.56,1.93)	1.00 (0.41,2.36)	HER2	1.08 (0.20,10.59)	0.22 (0.06,0.90)	0.48 (0.23,0.96)	
	0.72 (0.24,2.16)	0.69 (0.19,2.39)	0.69 (0.20,2.41)	mTOR	0.20 (0.02,1.51)	0.44 (0.05,2.23)	
	0.93 (0.15,6.11)	0.90 (0.13,6.30)	0.89 (0.13,6.17)	1.32 (0.15,11.13)	TKI	2.14 (0.57,7.77)	
	0.90 (0.61,1.31)	0.86 (0.40,1.75)	0.86 (0.41,1.77)	1.25 (0.39,3.94)	0.94 (0.15,6.17)	VEGF (R)	

Abbreviations: HER2, Human epidermal growth factor receptor-2; EGFR, epidermal growth factor receptor; VEGF(R), vascular endothelial growth factor (receptor); TKI, tyrosine kinase inhibitor; mTOR, mammalian target of rapamycin; HGRF, Hepatocyte growth factor receptor

### Ranking of molecules of targeted therapy

As was presented in Table 4, HER2 was the most effective drug with respect to OS while VEGF(R) invited most desirable results in terms of PFS, and both of them were associated with higher ORR. TKI turned out to be the most effective drug reducing the incidence of fatigue and diarrhoea, while EGFR was associated with less neutropenia events and thrombocytopenia compared with other treatments. VEGF(R) was effective in controlling the incidence of anaemia while m–TOR was associated with the least risk of vomiting.

**Table 4:** Surface under the cumulative rank curves value of different groups

	1-OS	2-OS	3-OS	1-PFS	2-PFS	3-PFS	ORR	Fatigue	Vomiting	Diarrhoea	Nausea	Anaemia	Thrombocytopenia	Neutropenia
All														
Placebo	0.231	0.290	0.356	0.265	0.318	0.287	0.151	0.750	0.424	0.881	0.571	0.538	0.683	0.851
EGFR	0.295	0.354	0.080	0.252	0.053	0.191	0.261	0.441	0.400	0.522	0.315	0.684	0.826	0.852
vHER2	0.737	0.751	0.808	0.721	0.720	0.659	0.765	0.278	0.398	0.117	0.460	0.240	0.409	0.433
MET	0.369	0.264	0.361	0.229	0.250	0.260	0.356	-	-	-	-	-	0.208	0.542
mTOR	0.510	0.538	0.589	0.732	0.784	0.811	0.732	0.353	0.692	0.176	0.524	0.232	0.153	0.074
TKI	0.605	0.628	0.619	0.424	0.469	0.427	0.594	0.980	0.499	0.838	0.535	0.512	0.657	0.293
VEGF(R)	0.752	0.675	0.687	0.877	0.906	0.866	0.640	0.197	0.588	0.466	0.594	0.795	0.566	rt0.456
Subgroup 1														
Placebo	0.361	0.463	0.451	0.416	0.415	0.378	0.297	0.870	0.452	0.976	0.668	0.353	0.748	0.548
EGFR	0.028	0.101	0.108	0.079	0.083	0.160	0.269	0.359	0.431	0.430	0.350	0.695	0.831	0.674
HER2	0.924	0.929	0.892	0.908	0.904	0.991	0.823	0.321	0.641	0.168	0.570	0.176	0.421	0.645
MET	0.430	0.308	0.399	0.288	0.323	0.244	0.501	0.207	0.225	-	-	-	-	-
VEGF(R)	0.757	0.699	0.650	0.810	0.776	0.727	0.609	0.450	0.477	0.425	0.412	0.775	0.293	0.408
Subgroup 2														
Placebo	0.037	0.055	0.175	0.032	0.006	0.098	0.063	0.671	0.443	0.792	0.550	0.653	0.481	0.932
EGFR	0.353	0.414	0.340	0.320	-	-	-	0.585	0.270	0.565	0.305	0.288	0.823	0.799
HER2	0.740	0.609	0.666	0.558	0.470	0.392	0.846	0.076	0.476	0.129	0.294	0.318	-	0.421
mTOR	0.409	0.450	0.500	0.781	0.738	0.794	0.594	0.438	0.672	0.303	0.547	0.400	0.070	0.071
TKI	0.527	0.572	0.546	0.311	0.289	0.295	0.407	0.939	0.476	0.766	0.559	0.579	0.540	0.415
VEGF(R)	0.936	0.901	0.774	0.999	0.997	0.921	0.589	0.291	0.663	0.444	0.746	0.763	0.586	0.362

Abbreviations: HER2, Human epidermal growth factor receptor-2; EGFR, epidermal growth factor receptor; VEGF(R), vascular endothelial growth factor (receptor); TKI, tyrosine kinase inhibitor; mTOR, mammalian target of rapamycin; HGRF, Hepatocyte growth factor receptor.

### Subgroup analysis

The results of subgroup one, which associated with first–line chemotherapy, were presented in Table 3, Supplementry Table 1 and Supplementry Figures 1, 2, while those of subgroup two, which associate with second-line chemotherapy were exhibited in Table 3, Supplementry Table 2 and Supplementry Figures 3, 4. In general, the results of subgroup analysis were consistent with previous results. However, HER2 used in combination with second-line chemotherapy did not seem to function as well as that with that in first-line chemotherapy, according to SUCRA.

### Publication bias

The estimate of publication bias was performed by the symmetry characteristics of the dots representing included trials with different colors in the funnel plots. According to Supplementry Figure 5, all of the funnel plots were focused in the triangle funnel areas in left and right directions, which suggested that the distribution of dots verified no significant publication bias or small study effect among ORR and all adverse events.

## DISCUSSION

In the present study, an NMA was performed to evaluate the safety and efficacy of different molecular targeted therapies including VEGF(R), EGFR, HER2, mTOR and TKI in combination with chemotherapy for patient with GC.

According to the results of this NMA, therapies targeting HER2 or VEGF(R) outperformed others with respect to survival outcomes and response rate, which was supported by previous studies A study conducted by Badiani *et al*. reported that patients treated with the above therapies exhibited significantly longer PFS, increased likelihood of disease control and enhanced ORR [32]. However, according our results, these two drugs were associated with higher risk of fatigue and diarrhea, which was also confirmed in other trials. For example, a study conducted by Bang *et al*. reported that HER2 invited more diarrhea and fatigue events [13], and another study designed by Fuchs *et al*. proved that VEGFR led to similar results [20]. Therefore, treatment recommendation should be made with caution.

Apart from VEGF(R) and HER2, agents targeting m-TOR also yielded desirable results in terms of survival and response rate, and similarly, it may lead to serious adverse events such as neutropenia and diarrhea. However, the role of m-TOR may need to be justified further since only one of the included studies covered the analysis of m-TOR, and the same with MET.

Although conducted as meticulously as possible, this NMA still has some limitations. First of all, it is inevitable that the reliability this study may be diluted by heterogeneity. For example, different chemotherapy treatments may results in different survival outcomes and adverse events. A study conducted by Koizumi *et al*. reported that the use of S-1 plus cisplatin yielded more satisfying results with respect to OS [8]. This is probably why the performance of HER2 in second-line chemotherapy was not as desirable as that in first-line chemotherapy. Secondly, we did not conduct a stratified or subgroup analysis due to the shortage of evidence. Some included studies did not report any information about tumor stage, type or specific dosage in the chemotherapy, which may weaken the reliability of corresponding results and conclusions. On top of that, we discovered that some of the eligible studies did not implement any blinding procedures. Thus, the quality of studies may be suspected and the overall effect size may be biased. Finally, our study did not include any approach to assessing the potential publication bias and we did not include any unpublished studies or articles in which the effect size may be different from that in ours.

However, despite all the limitations, our study contained several strengths with respect to study design and evidence synthesize. Unlike other meta-analysis or NMA that usually contains literatures in the last two decades, all of the included studies were carried out between 2010 and 2016, which contributed to the reliability and clinical significance of this article. A typical issue often arises from the fact that the control of confounding factors is lost when evidence is synthesized from individual studies. For instance, age is usually a significant risk factor in epidemiology. If confounding factors such as age are not controlled, the association between other risk factors and diseases may be significantly altered. The median age of GC patients in our study did not appear to be distributed in a wide range and this phenomenon may also reduce the degree of heterogeneity. Another major distinctive feature of our study came from the approach when evidence was synthesized. Unlike other meta-analysis or NMA, in which evidence was synthesized by the specific treatments, our study combined evidence based on a specific pathway of each target agents. This novel approach helped us determine which pathway is appropriate to be targeted in order to enhance the efficacy or safety of chemotherapy.

In summary, VEGF(R) and HER2 were the best two targeted therapies for GC, due to their high performance of efficacy outcomes whereas their adverse events should also be paid more attention. M-TOR may serve as alternative choice for its good performance with respect to survival outcomes, however, due to the lack of evidence, its role need to be further identified.

## MATERIALS AND METHODS

### Search strategy

We conducted the literature retrieval in PubMed, Embase and Cochrane Library systematically, aiming to select eligible articles which were designed as randomized controlled trials (RCTs) in GC patients. Following terms were used in the searching procedure: “vascular endothelial growth factor, vascular endothelial factor receptor, epidermal growth factor receptor, human epidermal growth factor receptor-2, mammalian target of rapamycin, tyrosine kinase inhibitor, chemotherapy, randomized controlled trials and gastric cancer”. The searching procedure was accomplished by two investigators independently. Reviews and duplicated studies were removed after scanning the titles and abstracts.

### Inclusion criteria

The following inclusion criteria were adopted: (1) studies must be RCTs; (2) targeted agents should be used in combination with chemotherapy; (3) patients should be diagnosed with GC; (4) studies should cover at least one of the included outcomes The articles were excluded according to the following rules: (1) treatments cannot form a network; (2) treatment withdrew; (3) treatment without former chemotherapy; Once the included study list was finalized, the Jadad Scale was used to assess the quality of included studies (Supplementary Table 3) [33].

### Data extraction

Two investigators independently searched the relevant data from eligible articles. In order to enhance the reliability and accuracy, a third party was involved to solve discrepancy. The following information is extracted: author, publication year, country, sample size, median age, target, treatments and outcomes, including OS and PFS of 1 year, 2 years and 3 years, ORR and 7 adverse events Among them, 1-year and 2-year survival were defined as short-term survival outcomes while 3-year and 5-year survival were long-term outcomes. Both survival outcomes and ORR were used to measure the relative efficacy of different target agents, while adverse events were used to measure their safety.

### Statistical analysis

In this NMA, the odds rates (ORs) with corresponding 95% credible intervals (CrIs) were utilized to measure response outcomes and adverse events. The hazard ratios (HRs) with 95% CrIs were used to assess the long-term prognostic factors outcomes. Furthermore, surface under the cumulative ranking curve (SUCRA) was calculated to illustrate the ranking probability of each treatment under different endpoint, with higher values indicating better efficacy or safety.

After the analysis of all trials, included studies were divided into two subgroups according to the chemotherapy, with subgroup one referring to first-line chemotherapy while subgroup two referring to second-line chemotherapy. These two subgroups were analyzed followed the methods above.

## SUPPLEMENTARY MATERIALS FIGURES AND TABLES



## Abbreviations

GC: Gastric cancer
VEGF: vascular endothelial growth factor
VEGFR: vascular endothelial factor receptor
HER2: human epidermal growth factor receptor 2
EGFR: Epidermal growth factor receptor
mTOR: mammalian target of rapamycin
TKI: Tyrosine kinase inhibitor
OS: overall survival
BSC: best support care
ORR: overall response rate
AEs: adverse effects
GI: gastrointestinal events
HE: hematological events
MA: meta-analyses
NMA: network meta-analysis
RCTs: randomized controlled trials
