# Supplementary material for: Efficacy and safety of different molecular targeted agents based on chemotherapy for gastric cancer patients treatment: a network meta-analysis
Source: Oncotarget. 2017 Apr 18;8(29):48253–62. doi: 10.18632/oncotarget.17192 (PMC5564643; doi:10.18632/oncotarget.17192)
Supplement: Supplementary file 1 [file oncotarget-08-48253-s001.pdf]

Efficacy and safety of different molecular targeted agents based on chemotherapy for gastric cancer patients treatment: a network meta-analysis

Supplementary Materials

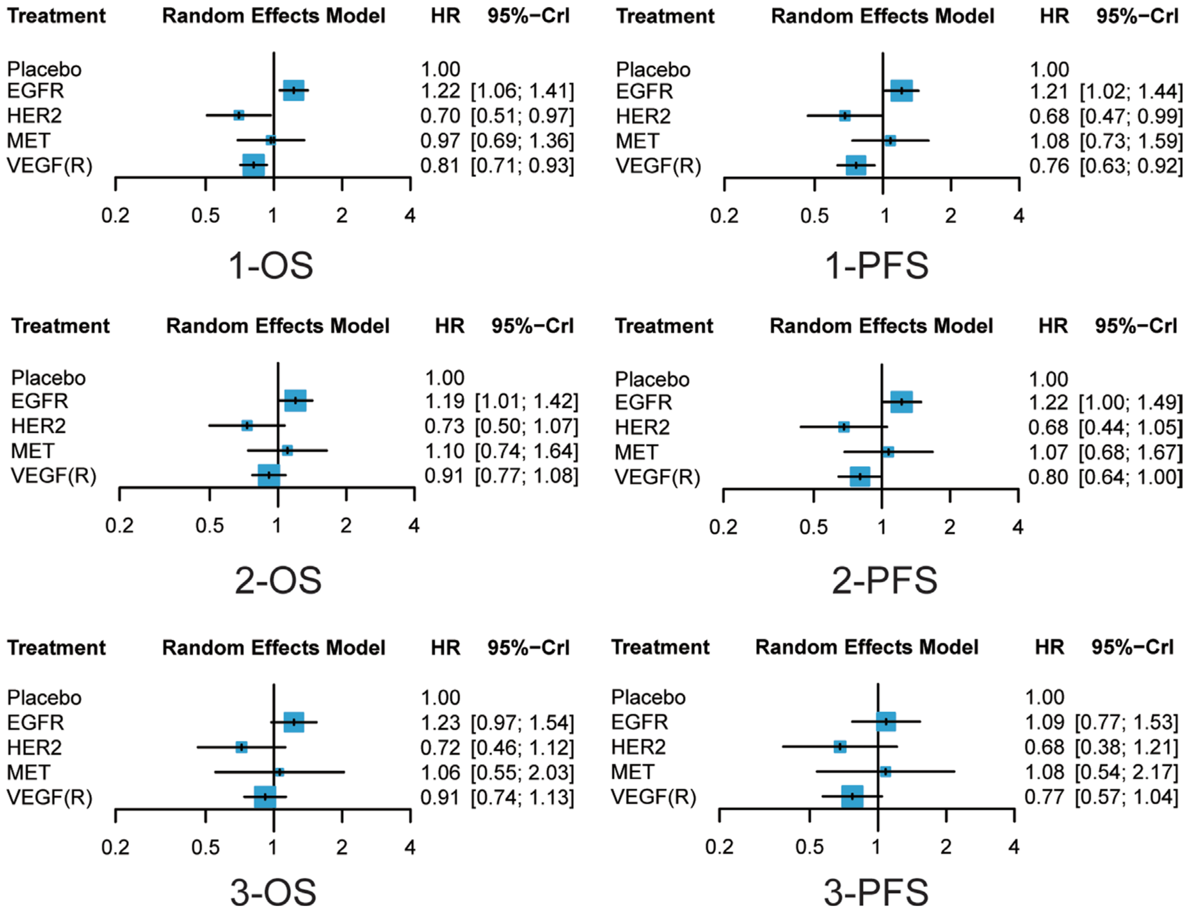

Supplementary Figure 1: Forest plots of survival outcomes in subgroup one. Hazard ratios (HRs) with corresponding 95% credible intervals (95% CrI) were used to measure the relative efficacy of different treatments.

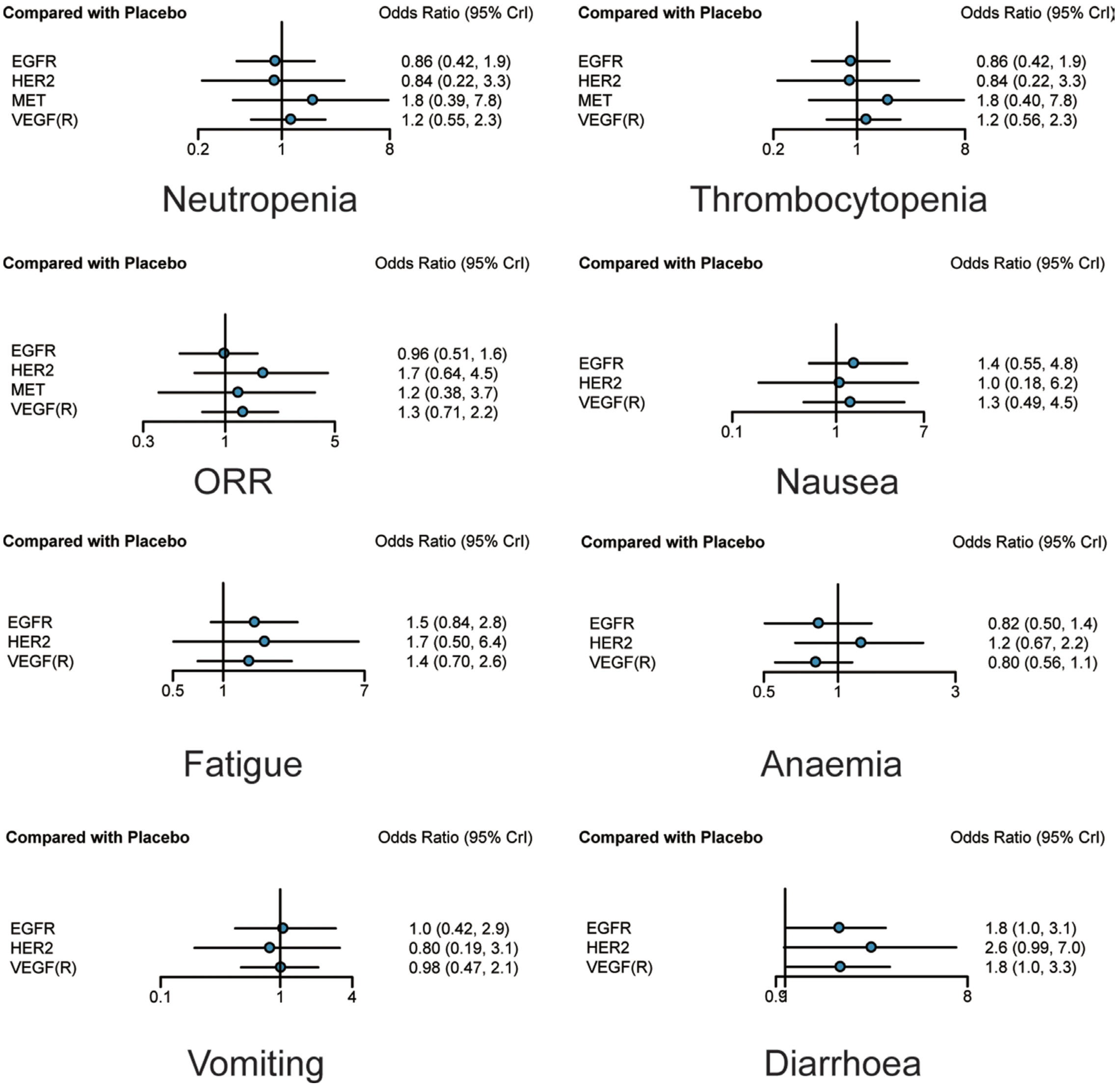

Supplementary Figure 2: Forest plots of overall response rate and adverse events in subgroup one. Odds ratios (ORs) with corresponding 95% credible intervals were used to measure the relative efficacy and safety of different treatments.

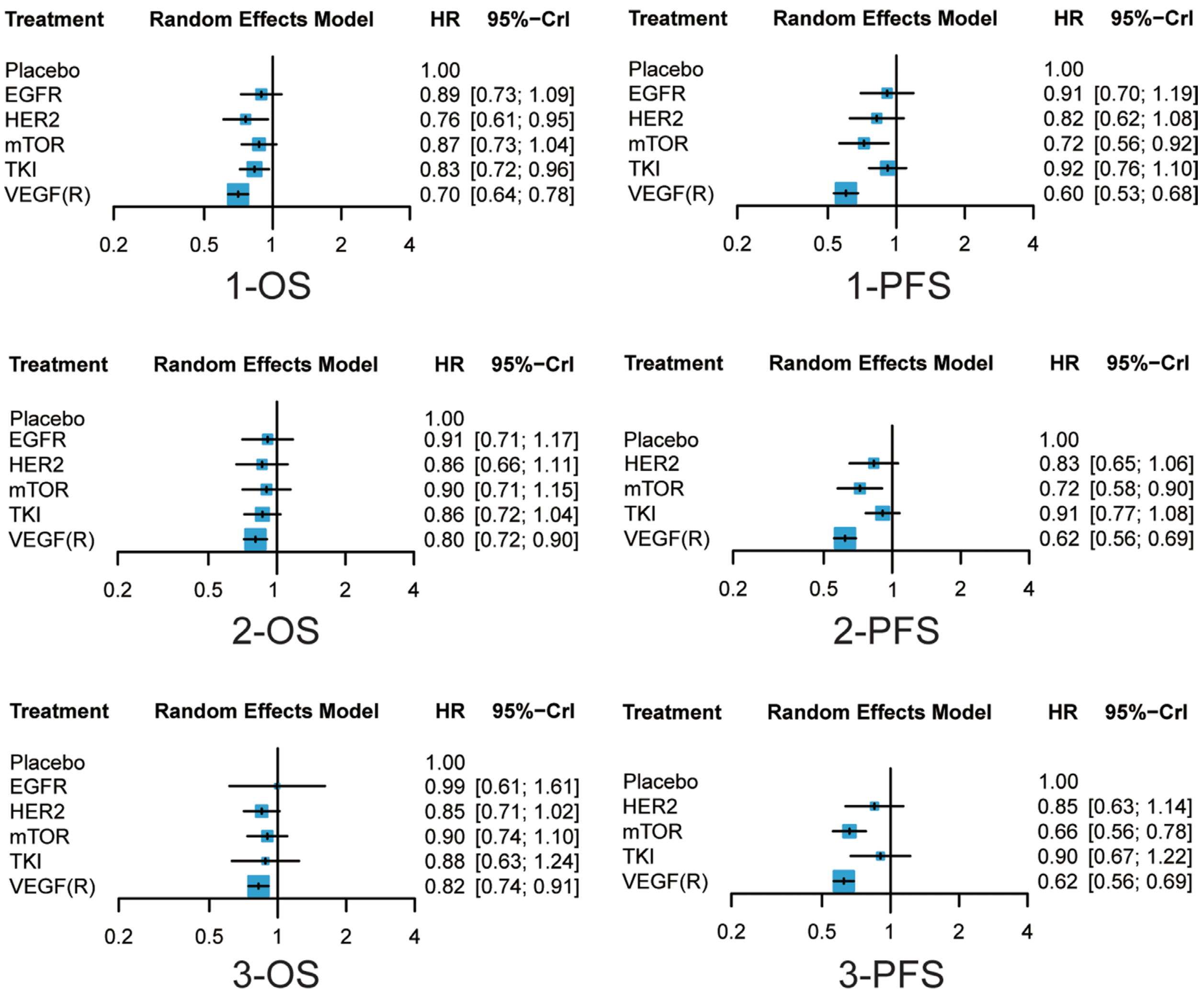

Supplementary Figure 3: Forest plots of survival outcomes in subgroup two. Hazard ratios (HRs) with corresponding 95% credible intervals (95% CrI) were used to measure the relative effiecacy of different treatments.

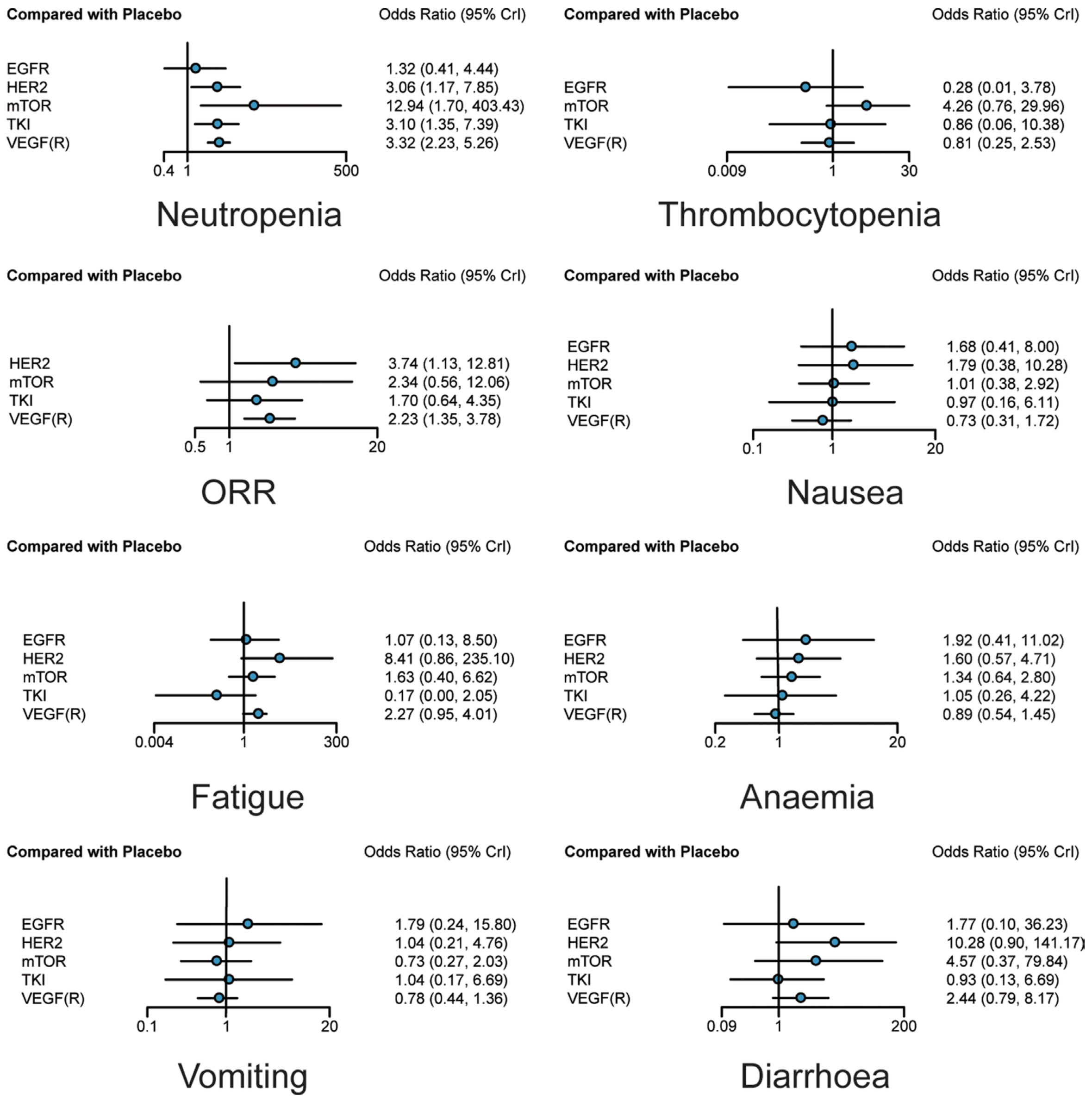

Supplementary Figure 4: Forest plots of overall response rate and adverse events in subgroup two. Odds ratios (ORs) with corresponding 95% credible intervals were used to measure the relative efficacy and safety of different treatments.

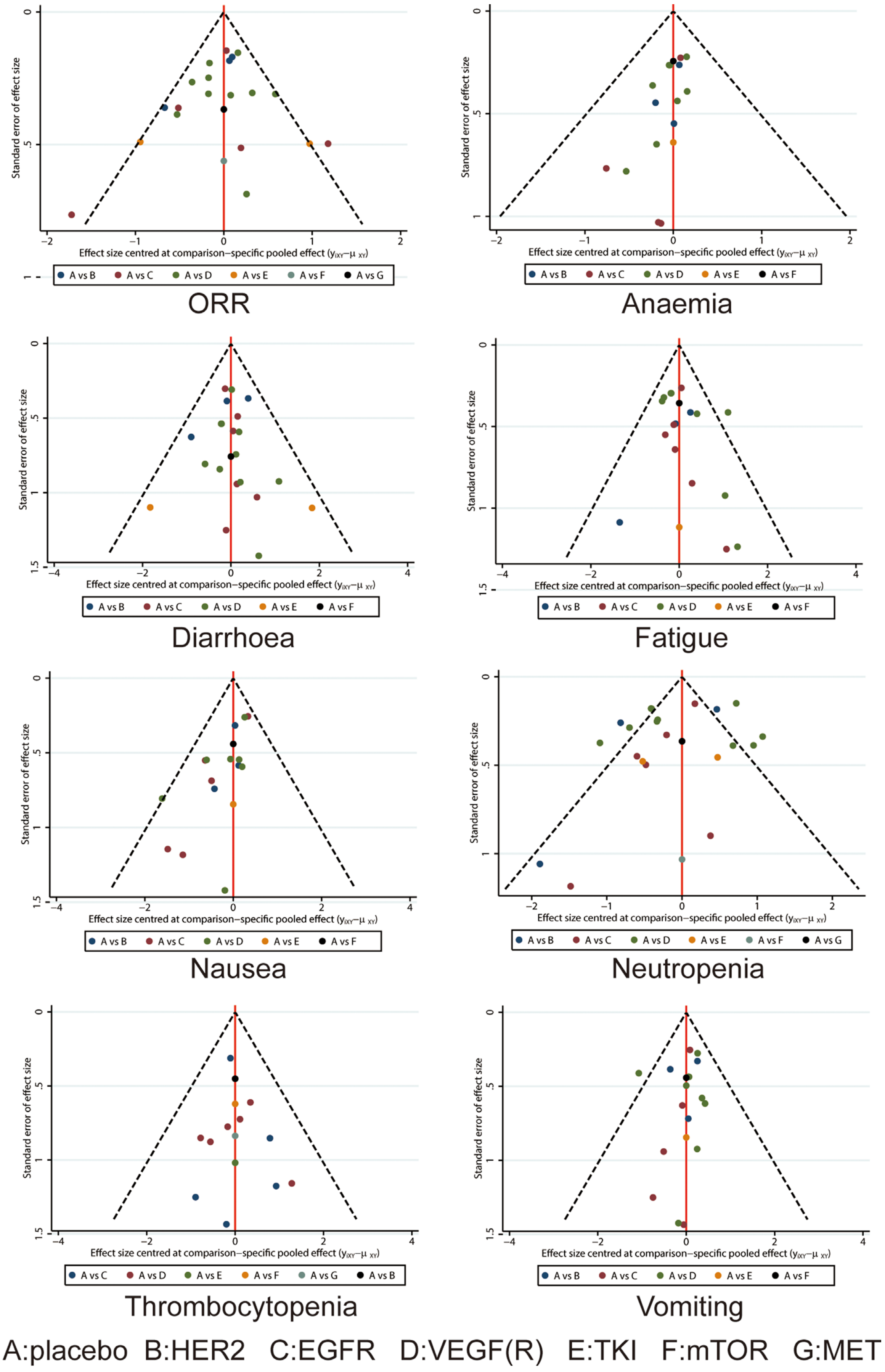

Supplementary Figure 5: Publication bias of survival outcomes, overall response rate and adverse events.

Supplementary Table 1: Network meta-analysis results of subgroup 1

**1-OS**	**Placebo**	1.21 (1.02,1.44)	0.68 (0.47,0.99)	1.08 (0.73,1.59)	0.76 (0.63,0.92)	**1-PFS**
	1.22 (1.06,1.41)	**EGFR**	0.56 (0.37,0.85)	0.89 (0.59,1.36)	0.63 (0.49,0.81)	
	0.70 (0.51,0.97)	0.57 (0.40,0.82)	**HER2**	1.59 (0.93,2.72)	1.12 (0.74,1.70)	
	0.97 (0.69,1.36)	0.79 (0.55,1.14)	1.39 (0.87,2.21)	**MET**	0.71 (0.46,1.08)	
	0.81 (0.71,0.93)	0.67 (0.55,0.81)	1.16 (0.82,1.65)	0.84 (0.58,1.21)	**VEGF (R)**	
**2-OS**	**Placebo**	1.22 (1.00,1.49)	0.68 (0.44,1.05)	1.07 (0.68,1.67)	0.80 (0.64,1.00)	**2-PFS**
	1.19 (1.01,1.42)	**EGFR**	0.56 (0.34,0.90)	0.88 (0.54,1.43)	0.66 (0.49,0.88)	
	0.73 (0.50,1.07)	0.61 (0.40,0.93)	**HER2**	1.57 (0.84,2.94)	1.18 (0.72,1.92)	
	1.10 (0.74,1.64)	0.92 (0.60,1.42)	1.51 (0.87,2.61)	**MET**	0.75 (0.46,1.23)	
	0.91 (0.77,1.08)	0.76 (0.60,0.97)	1.25 (0.82,1.89)	0.83 (0.54,1.28)	**VEGF (R)**	
**3-OS**	Placebo	1.09 (0.77,1.53)	0.68 (0.38,1.21)	1.08 (0.54,2.17)	0.77 (0.57,1.04)	**3-PFS**
	1.23 (0.97,1.54)	EGFR	0.63 (0.32,1.23)	0.99 (0.46,2.16)	0.71 (0.45,1.12)	
	0.72 (0.46,1.12)	0.59 (0.36,0.97)	HER2	1.59 (0.64,3.92)	1.13 (0.59,2.17)	
	1.06 (0.55,2.03)	0.87 (0.43,1.73)	1.47 (0.67,3.24)	MET	0.71 (0.33,1.52)	
	0.91 (0.74,1.13)	0.75 (0.55,1.02)	1.27 (0.78,2.08)	0.86 (0.44,1.71)	VEGF (R)	
**ORR**	**Placebo**	1.43 (0.55,4.81)	1.04 (0.18,6.17)	-	1.32 (0.49,4.53)	**Nausea**
	0.96 (0.51,1.60)	**EGFR**	0.73 (0.08,5.05)	-	0.93 (0.19,4.22)	
	1.70 (0.64,4.53)	1.77 (0.61,5.81)	**HER2**	-	1.26 (0.17,11.36)	
	1.19 (0.38,3.74)	1.23 (0.36,4.62)	0.70 (0.16,3.06)	**MET**	-	
	1.27 (0.71,2.16)	1.32 (0.62,3.03)	0.75 (0.24,2.27)	1.07 (0.30,3.74)	**VEGF (R)**	
**Neutropenia**	**Placebo**	0.86 (0.42,1.86)	0.84 (0.22,3.29)	1.75 (0.40,7.77)	1.16 (0.56,2.29)	**Thrombocytopenia**
	0.86 (0.42,1.88)	**EGFR**	0.98 (0.20,4.48)	2.05 (0.38,10.38)	1.35 (0.46,3.56)	
	0.84 (0.22,3.32)	0.99 (0.20,4.44)	**HER2**	2.10 (0.28,15.64)	1.38 (0.29,6.17)	
	1.77 (0.39,7.77)	2.05 (0.38,10.49)	2.10 (0.28,15.64)	**MET**	0.66 (0.12,3.39)	
	1.16 (0.55,2.32)	1.35 (0.46,3.56)	1.38 (0.29,6.17)	0.66 (0.12,3.42)	**VEGF (R)**	
**Fatigue**	**Placebo**	0.83 (0.51,1.36)	1.22 (0.67,2.23)	0.80 (0.55,1.14)		**Anaemia**
	1.51 (0.84,2.77)	**EGFR**	1.48 (0.68,3.19)	0.97 (0.53,1.79)		
	1.73 (0.51,6.42)	1.14 (0.30,4.81)	**HER2**	0.66 (0.33,1.31)		
	1.40 (0.70,2.56)	0.93 (0.36,2.10)	0.81 (0.18,3.13)	**VEGF (R)**		
**Vomiting**	**Placebo**	1.82 (1.00,3.16)	2.64 (0.99,7.03)	1.84 (1.02,3.29)		**Diarrhoea**
	1.03 (0.42,2.92)	**EGFR**	1.45 (0.48,4.62)	1.01 (0.45,2.32)		
	0.79 (0.19,3.16)	0.77 (0.13,3.86)	**HER2**	0.70 (0.22,2.12)		
	0.98 (0.47,2.08)	0.95 (0.26,3.00)	1.23 (0.26,6.17)	VEGF (R)		

Supplementary Table 2: Network meta-analysis results of subgroup 2

**1-OS**	**Placebo**	0.91(0.70,1.19)	0.82(0.62,1.08)	0.72(0.56,0.92)	0.92(0.76,1.10)	0.60(0.53,0.68)	**1-PFS**
	0.89(0.73,1.09)	**EGFR**	0.90(0.62,1.32)	0.79(0.55,1.14)	1.01(0.73,1.39)	0.66(0.49,0.88)	
	0.76(0.61,0.95)	0.85(0.63,1.16)	**HER2**	0.88(0.61,1.27)	1.12(0.80,1.55)	0.73(0.54,0.99)	
	0.87(0.73,1.04)	0.98(0.75,1.28)	1.14(0.86,1.52)	**mTOR**	1.27(0.93,1.73)	0.83(0.63,1.10)	
	0.83(0.72,0.96)	0.93(0.73,1.20)	1.09(0.84,1.43)	0.96(0.76,1.20)	**TKI**	0.66(0.52,0.82)	
	0.70(0.64,0.78)	0.79(0.63,0.99)	0.93(0.72,1.18)	0.81(0.66,0.99)	0.85(0.71,1.01)	**VEGF(R)**	
**2-OS**	**Placebo**	1.10(0.85,1.42)	0.83(0.65,1.06)	0.72(0.58,0.90)	0.91(0.77,1.08)	0.62(0.56,0.69)	**2-PFS**
	0.91(0.71,1.17)	**EGFR**	-	-	-	-	
	0.86(0.66,1.11)	0.95(0.66,1.36)	**HER2**	0.87(0.62,1.21)	1.09(0.81,1.47)	0.75(0.57,0.98)	
	0.90(0.71,1.15)	0.99(0.70,1.41)	1.05(0.73,1.49)	**mTOR**	1.26(0.95,1.67)	0.86(0.67,1.10)	
	0.86(0.72,1.04)	0.95(0.69,1.30)	1.00(0.73,1.38)	0.96(0.71,1.30)	**TKI**	0.68(0.56,0.84)	
	0.80(0.72,0.90)	0.88(0.67,1.17)	0.94(0.70,1.24)	0.89(0.68,1.17)	0.93(0.75,1.16)	**VEGF(R)**	
**3-OS**	**Placebo**	1.01(0.62,1.63)	0.85(0.63,1.14)	0.66(0.56,0.78)	0.90(0.67,1.22)	0.62(0.56,0.69)	**3-PFS**
	0.99(0.61,1.61)	**EGFR**	-	-	-	-	
	0.85(0.71,1.02)	0.86(0.51,1.43)	**HER2**	0.78(0.55,1.09)	1.06(0.70,1.62)	0.73(0.54,1.00)	
	0.90(0.74,1.10)	0.91(0.54,1.53)	1.06(0.81,1.39)	**mTOR**	1.37(0.97,1.93)	0.94(0.78,1.15)	
	0.88(0.63,1.24)	0.89(0.49,1.60)	1.04(0.71,1.52)	0.98(0.66,1.45)	**TKI**	0.69(0.50,0.95)	
	0.82(0.74,0.91)	0.83(0.51,1.36)	0.97(0.79,1.19)	0.91(0.73,1.14)	0.93(0.65,1.33)	**VEGF(R)**	
**ORR**	**Placebo**	1.68(0.41,8.00)	1.79(0.38,10.28)	1.01(0.38,2.92)	0.97(0.16,6.11)	0.73(0.31,1.72)	**Nausea**
	-	**EGFR**	1.05(0.12,9.78)	0.59(0.09,3.49)	0.57(0.05,5.58)	0.43(0.08,2.32)	
	3.74(1.13,12.81)	-	**HER2**	0.57(0.08,3.71)	0.54(0.04,5.99)	0.41(0.06,2.41)	
	2.34(0.56,12.06)	-	0.63(0.10,4.62)	**mTOR**	0.97(0.12,7.54)	0.72(0.18,2.66)	
	1.70(0.64,4.35)	-	0.45(0.09,2.05)	0.73(0.11,3.97)	**TKI**	0.76(0.10,5.26)	
	2.23(1.35,3.78)	-	0.59(0.16,2.25)	0.95(0.17,4.44)	1.31(0.45,3.97)	**VEGF(R)**	
**Neutropenia**	**Placebo**	0.28(0.01,3.78)	-	4.26(0.76,29.96)	0.86(0.06,10.38)	0.81(0.25,2.53)	**Thrombocytopenia**
	1.32(0.41,4.44)	**EGFR**	-	15.96(0.65,757.48)	3.13(0.08,194.42)	2.89(0.16,100.48)	
	3.06(1.17,7.85)	2.32(0.50,10.38)	**HER2**	-	-	-	
	12.94(1.70,403.43)	10.07(0.94,379.93)	4.35(0.45,151.41)	**mTOR**	0.19(0.01,4.22)	0.19(0.02,1.52)	
	3.10(1.35,7.39)	2.34(0.54,10.07)	1.00(0.29,3.67)	0.24(0.01,2.18)	**TKI**	0.94(0.06,16.28)	
	3.32(2.23,5.26)	2.51(0.70,8.85)	1.08(0.39,3.25)	0.26(0.01,2.05)	1.07(0.41,2.80)	**VEGF(R)**	
**Fatigue**	**Placebo**	1.92(0.41,11.02)	1.60(0.57,4.71)	1.34(0.64,2.80)	1.05(0.26,4.22)	0.89(0.54,1.45)	**Anaemia**
	1.07(0.13,8.50)	**EGFR**	0.84(0.12,5.53)	0.70(0.11,3.90)	0.55(0.06,4.35)	0.46(0.08,2.39)	
	8.41(0.86,235.10)	8.25(0.37,368.71)	**HER2**	0.84(0.23,2.94)	0.66(0.11,3.67)	0.55(0.17,1.72)	
	1.63(0.40,6.62)	1.54(0.12,18.73)	0.19(0.01,2.77)	**mTOR**	0.79(0.15,3.71)	0.66(0.27,1.60)	
	0.17(0.00,2.05)	0.16(0.00,3.97)	0.02(0.00,0.61)	0.10(0.00,1.72)	**TKI**	0.84(0.19,3.74)	
	2.27(0.95,4.01)	2.10(0.22,17.81)	0.26(0.01,2.75)	1.38(0.25,5.70)	12.94(0.93,518.01)	**VEGF(R)**	
**Vomiting**	**Placebo**	1.77(0.10,36.23)	10.28(0.90,141.17)	4.57(0.37,79.84)	0.93(0.13,6.69)	2.44(0.79,8.17)	**Diarrhoea**
	1.79(0.24,15.80)	**EGFR**	5.87(0.12,287.15)	2.69(0.05,146.94)	0.53(0.01,16.95)	1.39(0.06,31.82)	
	1.04(0.21,4.76)	0.56(0.04,7.32)	**HER2**	0.44(0.01,19.11)	0.09(0.00,2.16)	0.24(0.01,3.56)	
	0.73(0.27,2.03)	0.40(0.04,3.86)	0.70(0.11,4.66)	**mTOR**	0.20(0.01,5.00)	0.52(0.03,8.85)	
	1.04(0.17,6.69)	0.57(0.04,9.03)	1.02(0.09,11.13)	1.43(0.18,11.13)	**TKI**	2.61(0.28,26.84)	
	0.78(0.44,1.36)	0.43(0.05,3.53)	0.75(0.15,3.97)	1.07(0.33,3.39)	0.75(0.11,5.05)	**VEGF(R)**	

Supplementary Table 3: Jadad Scale of all included studies

Author	Year	Blinding	Randomization	Withdrawal/ dropouts
Shitara	2016	open	randomized	Reported
Hecht	2016	double	randomized	Reported
Muro	2016	open	randomized	Reported
Tebbutt	2016	open	randomized	Reported
Manish	2016	double	randomized	Reported
Markus	2016	double	randomized	Reported
Yoon	2016	double	randomized	Reported
Du	2015	open	randomized	Reported
Satoh	2015	double	randomized	Reported
Casak	2015	double	randomized	Reported
Shen	2015	double	randomized	Reported
Fuchs	2014	open	randomized	Reported
Satoh	2014	open	randomized	Reported
Wilke	2014	double	randomized	Reported
Xu	2014	open	randomized	Reported
Lordick	2013	open	randomized	Reported
Xu	2013	open	randomized	Reported
Ohtsu	2013	double	randomized	Reported
Richards	2013	open	randomized	Reported
Yi	2012	open	randomized	Reported
Ohtsu	2011	double	randomized	Reported
Bang	2010	open	randomized	Reported
Rao	2010	double	randomized	Reported
